# Effect of a text message intervention to reduce hazardous drinking among injured patients discharged from a trauma ward: a randomized controlled trial

**DOI:** 10.1038/s41746-018-0019-3

**Published:** 2018-05-02

**Authors:** Sarah Sharpe, Bridget Kool, Robyn Whittaker, Arier C. Lee, Papaarangi Reid, Ian Civil, Matthew Walker, Vanessa Thornton, Shanthi Ameratunga

**Affiliations:** 1 Population Health, Counties Manukau Health, Auckland, New Zealand; 2 Section of Epidemiology and Biostatistics, School of Population Health, University of Auckland, Auckland, New Zealand; 3 National Institute for Health Innovation, University of Auckland, and Waitemata District Health Board, Auckland, New Zealand; 4 Te Kupenga Hauora Māori, Faculty of Medical & Health Sciences, University of Auckland, Auckland, New Zealand; 5 Auckland City Trauma Service, Auckland City Hospital, Auckland, New Zealand; 6 Orthopaedic Department, Waitemata District Health Board, Auckland, New Zealand; 7 Emergency Department, Middlemore Hospital, Auckland, New Zealand

**Keywords:** Randomized controlled trials, Lifestyle modification

## Abstract

Screening and brief intervention for hazardous alcohol use in trauma care settings is known to reduce alcohol intake and injury recidivism, but is often not implemented due to resource constraints. Brief interventions delivered by mobile phone could overcome this challenge. This study aimed to evaluate the effect of a mobile phone text message intervention (YourCall^TM^) on hazardous drinkers admitted for an injury. The parallel two-group, single-blind, randomised controlled trial enrolled 598 injured patients aged 16–69 years identified as medium-risk drinkers at recruitment. The intervention group (*n* = 299) received 16 text messages incorporating brief intervention principles in the 4 weeks following discharge from hospital. Controls (*n* = 299) received usual care and one text message acknowledging participation in the trial. The primary outcome was the difference in hazardous alcohol use (assessed using AUDIT-C) between study groups at 3 months, with the maintenance of effect examined at 6 and 12 months’ follow-up. Data were analysed using a mixed-effects model for repeated measures. Both groups had similar baseline features. Compared to controls, hazardous drinking was significantly lower in the intervention group at 3 months and maintained over the 12-month follow-up period (least squares mean difference in AUDIT-C scores: −0.322; 95% CI: −0.636, −0.008; *p* = 0.04). The intervention effect was similar among Māori (New Zealand’s indigenous population) and non-Māori (interaction *p* = 0.59), and among younger (16–29 years) and older (30–69 years) patients (*p* = 0.77). The effectiveness of this intervention reflects the potential of low cost, scalable mobile health technologies to overcome common barriers in implementing alcohol harm reduction strategies following injury.

## Mobile health text message intervention to reduce hazardous drinking

Mobile phone text messages reduce dangerous patterns of alcohol consumption and could help prevent alcohol-related injuries. A trial carried out in New Zealand led by Sarah Sharpe at the University of Auckland examined the effect of sending 16 automated text messages over a four-week period to 299 adults discharged from hospital following an injury and identified as moderate risk hazardous drinkers. The messages, which encouraged patients to reduce their alcohol consumption and offered information on how to do so in an easy-to-understand and non-judgemental manner, reduced hazardous drinking throughout the 12-month follow-up period compared with a control group that received usual care. These findings suggest that text message interventions are an effective, low cost and easily scalable solution to curb harmful alcohol consumption.

## Introduction

Hazardous alcohol use is a leading risk factor for injury.^1–3^ Between 7–14% of all emergency department (ED) presentations,^4–6^ 8–60% of injury ED presentations^7^ and 23–50% of trauma centre admissions^8–10^ are reported to be alcohol-related. Prevention of alcohol-related trauma requires a multi-pronged public health approach including strategies that reduce access to and availability of alcohol, control sponsorship and advertising, drink-driving countermeasures, and appropriate interventions for hazardous drinkers.^11–13^

Screening for hazardous alcohol use and brief interventions (BI) in trauma care settings has been reported to reduce alcohol intake, injury recidivism and other alcohol-related harms.^8,14,15^ Despite inclusion in several guidelines,^16–18^ the implementation of BIs in busy clinical settings is challenged by time and resource constraints.^9,19–21^ Mobile phone (mHealth) text message approaches could contribute to reducing these barriers. Communicating via text messages is cost-effective, highly scalable, and has the potential to transform access to health promotion information and services due to the high uptake of mobile phones globally and the ubiquity of text messaging. Mobile phones have been referred to as 'the most accessible form of mediated communication in world history' and text messaging has become 'one of the most frequently used forms of mobile communication'.^22^

MHealth text message approaches show promise as an alternative delivery mode for alcohol BI.^23–26^ A randomised controlled trial of a 12-week text message alcohol intervention in 765 young-adult ED patients found small but significant decreases in binge-drinking days and the number of drinks consumed per drinking day in the intervention group compared with assessment and control groups at 3 months follow-up.^26^ A recently published Cochrane Collaboration systematic review of personalised digital interventions for reducing hazardous and harmful alcohol consumption in community-dwelling populations found moderate quality evidence that digital interventions lower alcohol consumption.^27^ The reviewers determined that there was insufficient information available to assess the impact of this mode of delivery on outcomes. Given the potential scalability and access to more disadvantaged communities, we consider the scant evidence relating to the impact of mHealth text message approaches (only one study^26^ in this review employed this mode of delivery) a particularly important research gap.

We developed a proactive, low-intensity, automated mobile phone text message programme drawing on BI principles (YourCall^TM^) designed to reduce hazardous drinking and alcohol-related harm among adults admitted to hospital following an injury. Following a feasibility study,^25^ we created, pre-tested, and refined the programme content. As described elsewhere,^28^ the programme was designed to be culturally relevant, appropriate, accessible, and engaging for Māori (New Zealand’s indigenous population) and Pacific audiences. The intervention consisted of a total of 16 text messages spread over a 4-week period and provided people the choice of three main language-pathways: (1) text messages in English with Te Reo Māori words of welcome and encouragement, (2) text messages in Te Reo Māori and (3) text message in English with an option to receive a greeting in Samoan, Tongan, Cook Island Māori, Niuean, Tokelauan, Tuvaluan or Fijian. The intervention length and frequency of text messages balanced the need to provide the core information elements of BI with a focus on keeping the frequency of messages to a minimum. This approach was informed by the feedback from participants during our feasibility study. Four text messages in the first week contained content that welcomed the recipient, gave feedback about their drinking, linked them to existing services (e.g. free-phone alcohol helpline) and encouraged contemplation about their drinking. The first text message in the second week contained an empathetic yet clear recommendation to cut down on drinking. This was followed during the second and third weeks by six messages focussed on providing information and tips or strategies about reducing alcohol consumption. The final three text message in the fourth week contained supportive and encouraging content with the key messages re-iterated.^28^

This trial aimed to evaluate the effect of the YourCall^TM^ text message BI (compared with usual care) in reducing hazardous drinking among adults admitted to hospital following an injury.

## Results

**Fig. 1:**
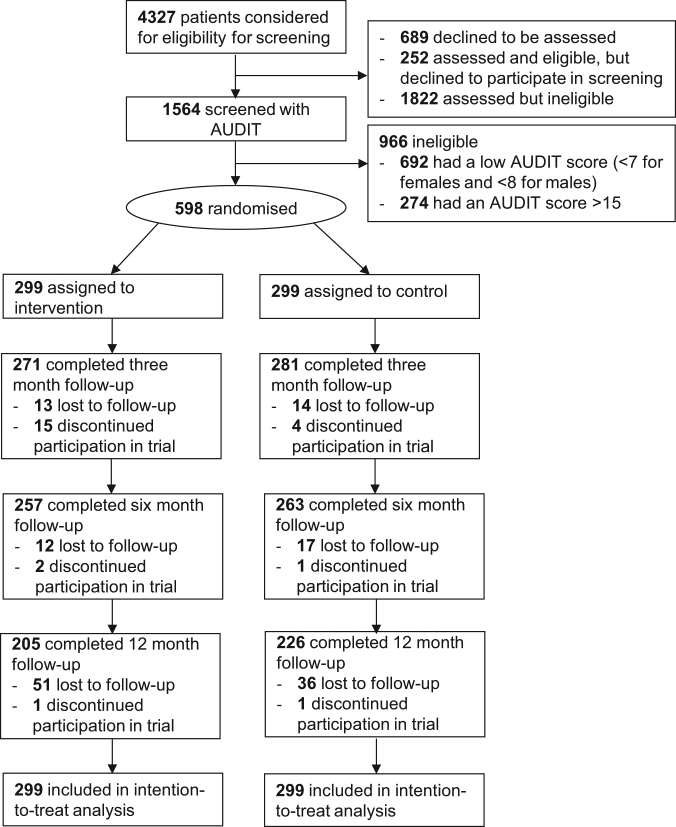
CONSORT flow diagram for YourCall trial

Participants were recruited from 9 November 2012 to 19 December 2013. Follow-up was completed by 2 February 2015. As outlined in the participant flow diagram (Fig. 1), 598 of the 1564 potentially eligible participants who were screened met the trial inclusion criteria. Of the 299 participants randomly allocated to the intervention group, 271 (91%), 257 (88%) and 205 (69%) provided data at 3, 6 and 12 months’ follow-up, respectively. Among the 299 participants in the control group, follow-up data at 3, 6 and 12 months were available for 281 (94%), 263 (88%) and 226 (76%), respectively.

Of 22 intervention group participants who texted back ‘stop’ during the intervention delivery period, 5 discontinued participation in the trial and one was lost-to follow-up. All participants (598) were included in intention-to-treat analyses.

### Baseline and drinking characteristics

Baseline characteristics of the two groups were similar with participants aged 16–29 years accounting for approximately half the trial participants and males accounting for almost 70% (Table 1). Twenty-one per cent of participants were Māori. Nearly 60% of participants drank alcohol ≥2 times per week, one third drank >6 drinks per typical drinking occasion and 37% drank ≥6 drinks per occasion weekly or more often.

**Table 1:** Baseline characteristics

Characteristics	Control group	Intervention group
	*n* = 299	*n* = 299
	*n* (%)^a^	*n* (%)^a^
Female	86 (28.8)	85 (28.4)
Age (mean, SD)	34 (13)	34 (13)
*Age groups* *(years)*		
16–29	144 (48.2)	145 (48.5)
30–69	155 (51.8)	154 (51.5)
*Ethnic groups*		
Māori	64 (21.4)	62 (20.7)
Pacific Peoples	34 (11.4)	42 (14.1)
Asian	15 (5.0)	12 (4.0)
NZ European & Other	186 (62.2)	183 (61.2)
*Hospital to which participant admitted*		
Middlemore Hospital	123 (41.1)	123 (41.1)
North Shore Hospital	87 (29.1)	87 (29.1)
Auckland City Hospital	89 (29.8)	89 (29.8)
*Employment*		
Employed	236 (78.9)	219 (73.2)
Student	31 (10.4)	40 (13.4)
Unemployed	13 (4.3)	18 (6.0)
Other	19 (6.4)	22 (7.4)
AUDIT-C score (mean, 95% CI)	6.82 (6.62–7.03)	6.87 (6.68–7.06)
*Drinking characteristics from AUDIT-C items*		
Drinks alcohol two or more times per week	178 (59.5)	174 (58.2)
Drinks more than six drinks per typical drinking occasion	100 (33.4)	101 (33.8)
Drinks six or more drinks per occasion weekly or more often	110 (36.8)	110 (36.8)
High volume of alcohol consumed per week typically^b^	102 (34.1)	104 (34.8)
*Current cigarette smoker*		
Yes	102 (34.1)	97 (32.4)
No	197 (65.9)	201 (67.2)
Unknown/refused to answer	0 (0)	1 (0.3)
*Current use of recreational drugs*		
Yes	59 (19.7)	64 (21.4)
No	239 (79.9)	233 (77.9)
Unknown/refused to answer	1 (0.3)	2 (0.7)
*Participant thinks their drinking played a role in the injury*		
Yes	41 (13.7)	42 (14.1)
No	258 (86.3)	257 (86.0)
*Participant thinks someone else’s drinking played a role in the injury*		
Yes	27 (9.0)	22 (7.4)
No	272 (91.0)	277 (92.6)
*Mechanism of injury*		
Fall	103 (34.5)	118 (39.5)
Struck by or against something	62 (20.7)	51 (17.1)
Cutting or piercing	57 (19.1)	45 (15.1)
Motor vehicle crash	36 (12.0)	39 (13.0)
Assault	9 (3.0)	10 (3.3)
Other	32 (10.7)	36 (12.0)
*Nature of injury* ^c^		
Lower limb (fractures, wounds, sprains)	132 (38.8)	131 (36.9)
Upper limb (fractures, wounds, sprains)	106 (31.2)	117 (33.0)
Other musculoskeletal	53 (15.6)	59 (16.6)
Head injuries	28 (8.2)	22 (6.2)
Internal (thoracic, abdominal, pelvic)	11 (3.2)	12 (3.4)
Other	10 (2.9)	14 (3.9)
*Intent of injury*		
Non-intentional	288 (96.3)	293 (98.0)
Intentional	9 (3.0)	5 (1.7)
Undetermined	2 (0.7)	1 (0.3)

AUDIT-C is Alcohol Use Disorders Identification Test-Consumption

^a^Number and % are provided, unless otherwise indicated

^b^Derived from combining AUDIT-C items 1 (i.e. frequency of drinking) and 2 (i.e. number of drinks consumed on a typical drinking occasion) to determine the number of drinks consumed per week typically, represented by 21 different categories or code pairs. In this analysis, high volume was defined as categories ‘5–6 drinks 2–3 times/week’, ‘7–9 drinks 2–3 times/week’, ’10 or more drinks 2–3 times/week’, ‘3–4 drinks 4 or more times/week’, ‘5–6 drinks 4 or more times/week’, 7–9 drinks 4 or more times/week’, and ’10 or more drinks 4 or more times/week’

^c^Participants could indicate one or more responses for these questions; therefore, values in each column do not add up to 100%

The percentage of participants with a non-hazardous drinking status measured using the short form of the Alcohol Use Disorders Identification Test (AUDIT-C score of <3 for females and <4 for males) increased from 0% at baseline to 9.9% in the control group and 13.4% in the intervention group at 3 months; 13.6% in the control group and 15.1% in the intervention group at 6 months, and 11.9% in the control group and 13.7% in the intervention group at 12 months (Table 2).

**Table 2:** Drinking characteristics of participants at baseline and follow-up time points

Drinking characteristics	Control group	Intervention group
	*n* (%)	*n* (%)
*Baseline*	*n* *=* *299*	*n* *=* *299*
Drinks alcohol two or more times per week^a^	178 (59.5)	174 (58.2)
Drinks more than six drinks per typical drinking occasion^b^	100 (33.4)	101 (33.8)
Drinks six or more drinks per occasion weekly or more often^c^	110 (36.8)	110 (36.8)
High volume of alcohol consumed per week typically^d^	102 (34.1)	104 (34.8)
Non-hazardous drinking status^e^	0 (0.0)	0 (0.0)
*3-month follow-up point*	*n* *=* *272*	*n* *=* *262*
Drinks alcohol two or more times per week	146 (53.7)	110 (42.0)
Drinks more than six drinks per typical drinking occasion	76 (27.9)	80 (30.5)
Drinks six or more drinks per occasion weekly or more often	79 (29.0)	66 (25.2)
High volume of alcohol consumed per week typically	81 (29.8)	60 (22.9)
Non-hazardous drinking status	27 (9.9)	35 (13.4)
*6-month follow-up point*	*n* *=* *250*	*n* *=* *245*
Drinks alcohol two or more times per week	124 (49.6)	120 (49.0)
Drinks more than six drinks per typical drinking occasion	59 (23.6)	43 (17.6)
Drinks six or more drinks per occasion weekly or more often	58 (23.2)	52 (21.2)
High volume of alcohol consumed per week typically	70 (28.0)	56 (22.9)
Non-hazardous drinking status	34 (13.6)	37 (15.1)
*12-month follow-up point*	*n* *=* *226*	*n* *=* *205*
Drinks alcohol two or more times per week	126 (55.8)	102 (49.8)
Drinks more than six drinks per typical drinking occasion	40 (17.7)	42 (20.5)
Drinks six or more drinks per occasion weekly or more often	66 (29.2)	54 (26.3)
High volume of alcohol consumed per week typically	55 (24.3)	44 (21.5)
Non-hazardous drinking status	27 (11.9)	28 (13.7)

^a^Derived from AUDIT-C item 1 'How often do you have a drink containing alcohol?'

^b^Derived from AUDIT-C item 2 'How many drinks containing alcohol do you have on a typical day when you are drinking?'

^c^Derived from AUDIT-C item 3 'How often do you have six or more drinks on one occasion?'

^d^Derived from combining AUDIT-C items 1 (i.e. frequency of drinking) and 2 (i.e. number of drinks consumed on a typical drinking occasion) to determine the number of drinks consumed per week typically, represented by 21 different categories or code pairs. In this analysis, high volume was defined as categories ‘5–6 drinks 2–3 times/week’, ‘7–9 drinks 2–3 times/week’, '10 or more drinks 2–3 times/week’, ‘3–4 drinks 4 or more times/week’, ‘5–6 drinks 4 or more times/week’, '7–9 drinks 4 or more times/week’, and '10 or more drinks 4 or more times/week’

^e^Non-hazardous drinking status is defined at the 3, 6 and 12-month follow-up points as an AUDIT-C score of <3 for females and <4 for males. At baseline, all participants were assessed as hazardous drinkers and this was an eligibility criterion for participation in the trial

**Fig. 2:**
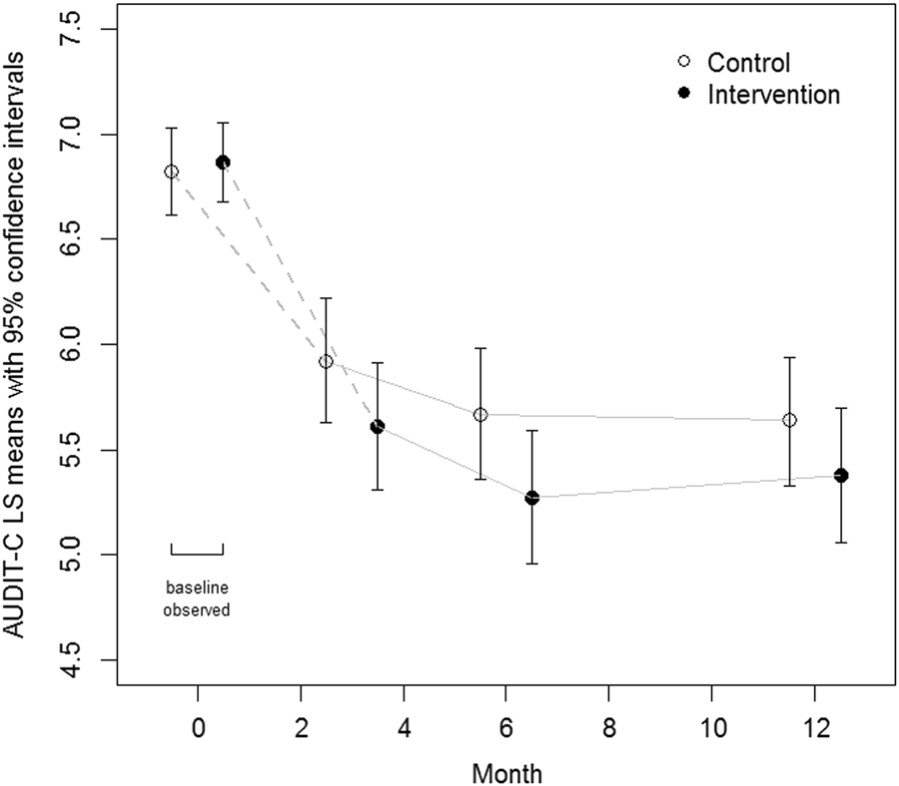
Least squares mean AUDIT-C scores at baseline and 3, 6 and 12-month follow-up points from a mixed model for repeated measures analysis

At baseline, observed mean AUDIT-C scores were 6.82 (95% CI 6.62–7.03) in the control group and 6.87 (95% CI 6.68–7.06) in the intervention group. During follow-up, reductions in hazardous alcohol use occurred in both groups (Fig. 2). Based on the mixed-effects models, the estimated mean AUDIT-C scores in the control group decreased to 5.92 (95% CI 5.63–6.22) at 3 months, 5.67 (95% CI 5.36–5.98) at 6 months and 5.64 (95% CI 5.33–5.94) at 12 months. In the intervention group, the equivalent scores were 5.61 (95% CI 5.31–5.91) at 3 months, 5.27 (95% CI 4.96–5.49) at 6 months and 5.38 (95% CI 5.06–5.70) at 12 months.

### Primary outcome

The mixed-effects model adjusted for age, sex, hospital, ethnicity and baseline AUDIT-C found the mean AUDIT-C score in the intervention group was on average 0.322 lower (95% CI −0.636, −0.008; *p* = 0.04) than the control group (Table 3). This effect was maintained across the 12-month follow-up period. Pre-planned secondary analysis revealed a non-significant interaction of treatment and ethnicity (*p* = 0.59), indicating the intervention effect was consistent among Māori and non-Māori. A post hoc analysis revealed the treatment effect was also similar among younger (16–29 years) and older (30–69 years) patients (interaction *p* = 0.77).

**Table 3:** Results of mixed modelling for the primary outcome

Fixed effect	Difference of least squares means			Type 3 tests	
	Estimate	95% CI		*F* value	Pr > *F*
AUDIT-C at baseline				113.17	<.001
*Treatment* (*Ref* = *Control*)				4.05	0.04
Intervention	−0.322	−0.636	−0.008		
*Time* (*Ref* = *3 months)*				6.19	0.002
6 months	−0.296	−0.474	−0.118		
12 months	−0.260	−0.463	−0.057		
Treatment×Time (Ref = Control, 3 months)				0.23	0.79
*Age Group (Ref 16–29)*				5.02	0.03
30–69	−0.360	−0.676	−0.044		
*Sex* (*Ref* = *male)*				1.41	0.24
Female	−0.218	−0.580	0.143		
*Hospital* (*Ref* = *Hospital 3)*				1.12	0.33
Hospital 1	−0.282	−0.662	0.098		
Hospital 2	−0.219	−0.626	0.188		
*Ethnic group* (*Ref* = *Non-Māori)*				2.44	0.12
Māori	−0.318	−0.719	0.081		

In the first per protocol sensitivity analysis, which excluded eight participants with a protocol violation (i.e. delay in commencing the text message intervention), the treatment effect was relatively unchanged. The estimated AUDIT-C score in the intervention group was on average 0.313 lower (95% CI −0.630, 0.005; *p* = 0.05) than the control group.

In the second per protocol sensitivity analysis which excluded 167 participants who were lost-to-follow-up or discontinued participation during the trial period, there was minimal difference in the treatment effect. The estimated AUDIT-C score in the intervention group was on average −0.335 lower (95% CI −0.673 to 0.004; *p* = 0.05) than the control group.

One serious adverse event was recorded during the study. This was the death of a participant, the cause of which (myocardial infarction) was unrelated to the trial. No adverse events were detected through daily reviews of the register of text-backs from participants. ‘Stop’ messages were sent by 22 participants (7% of intervention group).

## Discussion

This trial found that a low-intensity, automated, culturally appropriate, brief text message intervention, delivered to adults aged 16–69 years who had been admitted to hospital due to injury and screened positive for hazardous drinking of medium risk, led to a significant reduction in hazardous drinking in the intervention compared with control (usual care) group. This effect was maintained across the follow-up time points (3, 6 and 12 months) and was similar among Māori and non-Māori, and among younger (16–29 years) and older (30–69 years) participants.

In this trial, hazardous drinking was measured using the AUDIT-C score (range 0–12; score of ≥3 for females and ≥4 for males indicates hazardous drinking). The 0.3 lower AUDIT-C score on average in the intervention group compared with the control group equates to a 5% average reduction in score for the intervention group compared with controls, based on the mean AUDIT-C score in the control group at 3 months of 5.92. This effect size is comparable to the findings of previously published trials of face-to-face alcohol BIs^15,29,30^ and a text message alcohol intervention.^26^ Importantly, the YourCall^TM^ intervention effect was sustained throughout the 12-month follow-up period, a finding that differs from other studies which generally show a waning of effect over a year.^29^ This may reflect the fact that this mHealth intervention was delivered over a 4-week period with tailoring of messages to take account of days of the week when recreational drinking is likely to be more common.

While this trial was not designed to explain why the intervention was effective, there are several features that we consider may have contributed to this finding. Text messaging as a modality for BI may have advantages over traditional face-to-face BI as it is easily integrated into people’s lives using a familiar ‘every-day’ technology and in a way that is convenient and non-intrusive. For some people who are reluctant to access support through formal services, the anonymity provided by the automated service may have served as a positive characteristic. Providing a sequence of text messages over time may have resulted in a 'booster' effect. While we did not investigate different frequencies of text message delivery to assess dose effects, our approach with 16 text messages over 4 weeks appears to have delivered the necessary BI information components. In addition, the linkage of this intervention to a significant event (i.e. a hospitalisation due to an injury) was designed to use a ‘teachable moment’ when participants are more likely to consider this type of intervention helpful and timely. There are also important characteristics related to the carefully crafted content of the text messages, which were pre-tested with the target audience and key stakeholders, and refined during the development stage. The messages were intentionally simple and easy-to-understand, empathetic and non-judgemental in tone, and underwent cultural and language tailoring.

The participants in our trial were hazardous drinkers at medium risk of harm (AUDIT score 7–15 for women and 8–15 for men), they were not seeking help for alcohol issues, and the intervention was of very low intensity. While these characteristics may result in an underestimation of the potential effect of mobile health interventions on all problem drinkers, BIs are treatments designed specifically for medium-risk drinkers rather than drinkers at higher risk of harm and dependent on alcohol.^31^ In medium-risk groups, low intensity or ‘very brief’ interventions are reported to be just as effective as more intensive interventions.^15,29,30^

The similar treatment effect among Māori and non-Māori is of particular importance in the New Zealand context. Māori people experience disproportionate harm from alcohol compared with other ethnic groups.^32^ Given the burden of comprehensive health inequities borne by Māori, interventions must be shown to be equally effective for Māori in order to ensure that these efforts do not unwittingly increase inequity. Our focus on developing culturally appropriate content that would engage Māori people and be relevant to Māori lived realities may have contributed to the equivalence of treatment effects. A previous trial evaluating an mHealth smoking cessation intervention which incorporated Māori-specific test messages found the intervention was as effective for Māori as non-Māori at increasing quit rates.^33^

The effect of alcohol screening on study groups, as seen in the reduction in mean AUDIT-C in the control group in this study, has been noted in other studies.^34^ Reasons for this observation could include an effect from the screening/assessment process on hazardous drinking,^35^ regression to the mean^36^; the effect of being unwell with an injury and/or recovering from surgery, therefore not taking part in usual activities; and the influence of participating in a research study. The trial design, however, gauged the impact of the intervention, over and above these potential phenomena.

The strengths of this randomised controlled trial include its large size, good follow-up rates at 3 months, broad age range, generalisability to adult inpatient trauma care patients (regardless of whether alcohol played a role in the injury), focus on medium-risk drinkers (a previously neglected group), and recruitment practices that ensured participation of Māori patients (21% of study participants; 9% of the Auckland Region population aged 15–69 years^37^).

The study findings, however, must also be interpreted in light of limitations, particularly the differential loss to follow-up (31% and 24% in the intervention and control groups at 12 months). The sensitivity analysis excluding participants who were lost-to-follow-up or discontinued their participation was reassuring in that the treatment effect was relatively unchanged. The larger proportion of participants lost from the intervention group may be partly explained by the more frequent texts, and therefore prompts that this group had to text back ‘stop’. This was activated by 22 participants although only six discontinued participation or were lost-to follow-up.

Self-reported drinking measures are known to be susceptible to measurement bias^38^ as people tend to under-report the frequency and quantity of drinking. As we do not expect the level of under-reporting to be different in the two groups, we do not expect this to threaten the validity of the intervention effect. However, given the lack of sufficient power to undertake more detailed sub-group analyses, we cannot rule out possible masking of different effects in subgroups that we have not examined. Aspects not explored in this study but worthy of future research include the specific intervention elements that account for its effectiveness, levels of interactivity or booster doses that could enhance benefits, and reasons why some participants elected to ‘stop’ the messages.

The findings of this trial provide further evidence to support the emerging literature about the effectiveness of text message interventions designed to reduce hazardous drinking. While the absolute effects are likely to be modest, these could have important effects at the population level. As a delivery mode, mHealth strategies expand the options available to healthcare services to provide low cost, highly scalable, time-saving interventions. These may particularly appeal to patients given the convenience of access, integration into daily life, cultural appropriateness and technological engagement. With high and expanding mobile phone coverage worldwide, these aspects make mHealth interventions for hazardous alcohol use particularly salient in economically disadvantaged groups and low- and middle-income countries.

Further research should address the barriers that can impede the implementation of screening and BI, including mHealth options, into every-day practice in healthcare settings, including trauma care. This is critical to translating research evidence to best practice in ‘real world’ settings.

In summary, compared with usual care, the YourCall™ intervention (a low-intensity mHealth text message programme using BI principles) resulted in a significant reduction in hazardous drinking among patients admitted following an injury. The intervention effect (in terms of mean group differences) was sustained over the 12-month follow-up period and similar in Māori and non-Māori participants. MHealth interventions are scalable, low-cost approaches that could overcome barriers to implementing BIs in clinical settings.

## Methods

### Study design

We conducted a simple, two-group, parallel, randomised controlled trial to evaluate the ‘YourCall’ intervention, the protocol for which has been published.^39^ The methods were performed in accordance with relevant regulations and guidelines. The trial was approved by the New Zealand Health and Disability Ethics Committee (12/NTB/28), and was registered with the Australian New Zealand Clinical Trials Registry (anzctr.org.au; Identifier: ACTRN12612001220853).

### Participants

Participants were inpatients aged 16 to 69 years admitted for an injury-related cause to the three trauma-admitting hospitals (North Shore, Auckland City, and Middlemore) in Auckland, New Zealand’s largest city (population 1.4 million). In order to be eligible, they had to be current drinkers, use a mobile phone which was not shared with someone else, be able to read and send text messages, be able to complete surveys in English, be expected to be discharged home, and be competent to provide informed consent. During recruitment (March 2013), two eligibility criteria were broadened to increase the number of potentially eligible participants. The upper age limit was increased from 60 to 69 years and the initial restriction of only including people admitted for 24 h or more was replaced with including all hospital admissions regardless of length of stay. Pregnant women, tourists and patients with self-harm injuries were excluded.

Using procedures described in detail previously,^39^ eligible patients were identified, information about the study was provided, and written informed consent was obtained from those interested in participating in the trial. Study participants were then screened for hazardous drinking using the Alcohol Use Disorders Identification Test (AUDIT).^40^

Patients were included in the trial if they were considered to be at medium risk of alcohol problems (AUDIT scores: 7–15 for females; 8–15 for males). Patients with higher scores were excluded as the appropriate management involves counselling, specialist evaluation and treatment.^31^

### Randomisation and masking

Trial participants were randomly assigned by computer to receive the ‘YourCall’ intervention or a control programme (usual care). Computer-based randomisation ensured balance in treatment assignment for randomisation factors including age (16–29 years, 30–69 years), sex, ethnicity (Māori, non-Māori) and recruitment hospital. Due to the nature of the intervention only single blinding was possible (i.e. researchers only). Research assistants (blind to treatment allocation) enrolled participants, undertook all baseline data collection and initiated the computer-based randomisation procedure for each participant at the time of their discharge from hospital.

### Procedures

All participants received an information brochure (*The straight up guide to standard drinks*^41^) at the time of enrolment. Those allocated to the intervention group received the ‘YourCall’ program’s 16 text messages sent over 4 weeks, starting 7–10 days after discharge from hospital.^28,39^ Control group participants received one text message following discharge from hospital. This message acknowledged their participation in the trial and indicated they would be contacted in 3 months’ time.

Baseline assessments included collection of demographic data and screening for hazardous alcohol use using the AUDIT. Follow-up self-reported assessments were conducted at 3, 6, and 12 months. At 3 and 6 months, questions were delivered via text message with participants responding via text. Responses were recorded automatically in the data management system. Participants were invited to complete an online survey at the 12-month time point. Those not responding at the follow-up points were contacted by phone by research assistants and assessments were conducted via telephone.

### Outcomes

The primary trial outcome was the difference in hazardous alcohol use between the intervention and control groups at 3 months, with the maintenance of effect examined at 6 and 12 months. Hazardous alcohol use at follow-up was assessed using the AUDIT-C tool.^42^ This comprises the first three questions of the 10-item AUDIT, scored on a scale of 0–12. The AUDIT-C tool is known to have sound psychometric properties^42,43^ and has been validated for identification of hazardous alcohol use in a range of settings including with admitted trauma patients^44^ and online with adults seeking help for their drinking.^45^ The tool has favourable test-retest reliability, including over one and three month intervals,^43^ and allows the accurate monitoring of patients’ risk over time.^42^ Despite the common use of AUDIT-C in research studies including in online formats,^23,46^ the instrument has not, to our knowledge, been formally validated for use via text message nor been delivered via text message in other published studies for follow-up purposes.

Serious adverse events reported by participants or next-of-kin were recorded. At enrolment, participants were given information on texting-back ‘stop’ at any time if they did not wish to receive further ‘YourCall’ text messages. A register of unsolicited text-backs from participants was reviewed daily with responses guided by the study protocol.

### Statistical analysis

A sample size of at least 570 was expected to provide 80% power, at the 0.05 level of significance and with 70% follow-up, to detect a true difference of 0.5 (7.5%) between the intervention and control groups in their mean 3-month AUDIT-C scores.

Baseline demographic variables (age, sex, ethnic group), employment and education, mobile phone usage, cigarette smoking and recreational drug usage, self-reported role of alcohol in the injury, nature of injury, and AUDIT-C mean scores were summarised.^39^

AUDIT-C scores at 3, 6 and 12 months were analysed using the mixed-effects model for repeated measures. Treatment group, visit, group and visit interaction, the randomisation variables of age, gender, ethnicity and hospital were assessed as fixed effects, baseline AUDIT-C measure as a covariate, and participant as a random effect in the mixed-effect model.^39^ The primary outcome was determined by the treatment effect at 3 months. An unstructured variance (co)variance structure was used to model the within-subject error. The Kenward–Roger method was used to estimate the denominator degrees of freedom for fixed effects.

To assess the effectiveness of the programme for Māori and non-Māori, the analysis of the primary outcome was repeated with treatment and ethnicity (Māori vs non-Māori) interaction added to the model.^39^ A post hoc interaction analysis also examined if the treatment effect varied by age group, given the suggestion that some BIs for alcohol use are less effective among youth. As the study was not powered to test for these interactions, the results need to be interpreted with caution.

Data were analysed following a pre-specified analysis plan. All analyses were performed using SAS version 9.4 (SAS Institute Inc., Cary, NC). All statistical tests were two-tailed and a 5% significance level maintained throughout. All evaluations were performed on the ‘intention to treat’ principle, i.e. participants were analysed in the group they were randomised regardless of whether they were withdrawn or there was a protocol deviation. No adjustments for multiplicity were made for any of the outcomes. No imputations were made for missing data.

Two per protocol analyses were also performed on the primary outcome as sensitivity analyses. In the first, the per protocol population consisted of all randomised participants excluding eight participants who had a protocol violation due to an intervention commencement delay of more than 2 weeks. The per protocol population in the second sensitivity analysis comprised all randomised participants excluding the 167 participants who were lost-to-follow-up or discontinued their participation during the trial.

### Data availability

Due to the conditions of the informed consent obtained from participants, the institutional and Ministry of Health ethical requirements do not permit us to share participant data from this study.

## References

[1] Rehm J (2009). Global burden of disease and injury and economic cost attributable to alcohol use and alcohol-use disorders. Lancet.

[2] Taylor B (2010). The more you drink, the harder you fall: a systematic review and meta-analysis of how acute alcohol consumption and injury or collision risk increase together. Drug Alcohol Depend..

[3] Room R, Babor T, Rehm J (2005). Alcohol and public health. Lancet.

[4] Stewart R (2014). The impact of alcohol-related presentations on a New Zealand hospital emergency department. NZ Med. J..

[5] McDonald AJ, Wang N, Camargo CA (2004). US emergency department visits for alcohol-related diseases and injuries between 1992 and 2000. Arch. Intern. Med..

[6] Egerton-Warburton D, Gosbell A, Wadsworth A, Fatovich DM, Richardson DB (2014). Survey of alcohol-related presentations to Australasian emergency departments. Med. J. Aust..

[7] Cherpitel CJ (2007). Alcohol and injuries: a review of international emergency room studies since 1995. Drug Alcohol Rev..

[8] Gentilello LM (1999). Alcohol interventions in a trauma center as a means of reducing the risk of injury recurrence. Ann. Surg..

[9] Hosking J (2007). Screening and intervention for alcohol problems among patients admitted following unintentional injury: a missed opportunity?. NZ Med. J..

[10] Deutch SR (2004). Drug and alcohol use among patients admitted to a Danish trauma centre: a prospective study from a regional trauma centre in Scandinavia. Eur. J. Emerg. Med..

[11] Babor, T. *Alcohol: No Ordinary Commodity: Research and Public Policy* (Oxford University Press: Oxford, 2010).

[12] Anderson P, Chisholm D, Fuhr DC (2009). Effectiveness and cost-effectiveness of policies and programmes to reduce the harm caused by alcohol. Lancet.

[13] World Health Organization. (2010). Global Strategy to Reduce the Harmful Use of Alcohol.

[14] Nilsen P (2008). A systematic review of emergency care brief alcohol interventions for injury patients. J. Subst. Abuse Treat..

[15] Schmidt CS (2016). Meta-analysis on the effectiveness of alcohol screening with brief interventions for patients in emergency care settings. Addiction.

[16] American College of Surgeons Committee on Trauma. Alcohol Screening and Brief Intervention (SBI) for Trauma Patients. Committee on Trauma Quick Guide (ASCOT: Chicago, 2007).

[17] National Institute on Alcohol Abuse and Alcoholism. Helping Patients Who Drink Too Much. A Clinician’s Guide. Updated 2005 Edition (U.S. Department of Health & Human Sciences: Bethesda, 2007).

[18] American College of Emergency Physicians. *Alcohol Screening and Brief Intervention in the Emergency Department*. https://www.acep.org/Clinical---Practice-Management/Alcohol-Screening-and-Brief-Intervention-in-the-ED/ (2016).

[19] Cunningham RM (2010). National survey of emergency department alcohol screening and intervention practices. Ann. Emerg. Med..

[20] Cryer HG (2005). Barriers to interventions for alcohol problems in trauma centers. J. Trauma.

[21] Schermer CR (2003). National survey of trauma surgeons’ use of alcohol screening and brief intervention. J. Trauma.

[22] Hall AK, Cole-Lewis H, Bernhardt JM (2015). Mobile text messaging for health: a systematic review of reviews. Annu. Rev. Public Health.

[23] Suffoletto B, Callaway C, Kristan J, Kraemer K, Clark DB (2012). Text-message-based drinking assessments and brief interventions for young adults discharged from the emergency department. Alcohol Clin. Exp. Res..

[24] Crombie, I. K. et al. Reducing alcohol-related harm in disadvantaged men: development and feasibility assessment of a brief intervention delivered by mobile telephone. *Public Health Res.* **1**, 1–158 (2013). 27466658

[25] Kool, B., Smith, E., Raerino, K. & Ameratunga, S. Perceptions of adult trauma patients on the acceptability of text messaging as an aid to reduce harmful drinking behaviours. *BMC Res. Notes* **7**. 10.1186/1756-0500-7-4 (2014). 10.1186/1756-0500-7-4 PMC3884009 24387293

[26] Suffoletto B (2014). A text message alcohol intervention for young adult emergency department patients: a randomized clinical trial. Ann. Emerg. Med..

[27] Kaner EF (2017). Personalised digital interventions for reducing hazardous and harmful alcohol consumption in community-dwelling populations. Cochrane Database Syst. Rev..

[28] Sharpe S (2015). Development of a text message intervention aimed at reducing alcohol-related harm in patients admitted to hospital as a result of injury. BMC Public Health.

[29] Moyer A, Finney JW, Swearingen CE, Vergun P (2002). Brief interventions for alcohol problems: a meta-analytic review of controlled investigations in treatment-seeking and non-treatment-seeking populations. Addiction.

[30] Kaner EF (2009). The effectiveness of brief alcohol interventions in primary care settings: a systematic review. Drug Alcohol Rev..

[31] Babor TF, Higgins-Biddle JC (2001). Brief Intervention for Hazardous and Harmful Drinking. A Manual for Use in Primary Care.

[32] Meiklejohn J, Connor J, Kypri K (2012). One in three New Zealand drinkers reports being harmed by their own drinking in the past year. NZ Med. J..

[33] Bramley D (2005). Smoking cessation using mobile phone text messaging is as effective in Maori as non-Maori. NZ Med. J..

[34] Kypri K, Langley JD, Saunders JB, Cashell-Smith ML (2007). Assessment may conceal therapeutic benefit: findings from a randomized controlled trial for hazardous drinking. Addiction.

[35] McCambridge J, Butor-Bhavsar K, Witton J, Elbourne D (2011). Can research assessments themselves cause bias in behaviour change trials? A systematic review of evidence from solomon 4-group studies. PLoS ONE.

[36] McCambridge J, Kypri K, McElduff P (2014). Regression to the mean and alcohol consumption: a cohort study exploring implications for the interpretation of change in control groups in brief intervention trials. Drug Alcohol Depend..

[37] Statistics New Zealand. *2013 Census Quickstats about Māori*. www.stats.govt.nz (2013).

[38] Kypri K (2007). Methodological issues in alcohol screening and brief intervention research. Subst. Abus..

[39] Ameratunga S (2017). Effectiveness of the YourCall text message intervention to reduce harmful drinking in patients discharged from trauma wards: protocol for a randomised controlled trial. BMC Public Health.

[40] Babor TF, Higgins-Biddle JC, Saunders JB, Monteiro MG (2001). The Alcohol Use Disorders Identification Test. Guidelines for Use in Primary Care.

[41] Alcohol Advisory Council of New Zealand (ALAC). *Straight Up Guide to Standard Drinks*. https://www.hpa.org.nz/sites/default/files/imported/field_publication_file/StdDrinks2006.pdf (2006).

[42] Bradley KA (2007). AUDIT-C as a brief screen for alcohol misuse in primary care. Alcohol Clin. Exp. Res..

[43] Reinert DF, Allen JP (2007). The alcohol use disorders identification test: an update of research findings. Alcohol Clin. Exp. Res..

[44] Vitesnikova J, Dinh M, Leonard E, Boufous S, Conigrave K (2014). Use of AUDIT-C as a tool to identify hazardous alcohol consumption in admitted trauma patients. Injury.

[45] Khadjesari Z (2017). Validation of the AUDIT-C in adults seeking help with their drinking online. Addict. Sci. Clin. Pract..

[46] Kypri K (2013). Web-based alcohol intervention for Maori university students: double-blind, multi-site randomized controlled trial. Addiction.

